# Skin Deep and Beyond: Unravelling B Cell Extracellular Matrix Interactions in Cutaneous Immunity and Disease

**DOI:** 10.1111/exd.70068

**Published:** 2025-03-06

**Authors:** Rebecca Diehl, Stefanie Hübner, Saskia Lehr, Marta Rizzi, Kilian Eyerich, Alexander Nyström

**Affiliations:** ^1^ Department of Dermatology University Medical Center Freiburg, Faculty of Medicine, University of Freiburg Freiburg Germany; ^2^ Center of Chronic Immunodeficiency CCI University Clinics and Medical Faculty Freiburg Germany; ^3^ CIBSS ‐ Centre for Integrative Biological Signalling Studies University of Freiburg Freiburg Germany; ^4^ Division of Clinical and Experimental Immunology, Institute of Immunology, Center for Pathophysiology, Infectiology and Immunology Medical University of Vienna Vienna Austria; ^5^ Department of Rheumatology and Clinical Immunology University Medical Center Freiburg, Faculty of Medicine, University of Freiburg Freiburg Germany

## Abstract

The extracellular matrix (ECM) is a crucial component in multicellular organisms, serving as both a structural scaffold and active signalling units. While the role of the ECM, namely, to maintain homeostasis and steer adaptive immunity, is well described in secondary lymphoid organs, it is underappreciated in the skin—despite remarkable molecular similarity. Here, we examine how the specialised organisation of the ECM influences B cell development and function in both skin and secondary lymphoid organs with a special focus on ECM–integrin signalling. We discuss the presence and function of B cells in healthy and diseased skin, including their role in wound healing, autoimmune responses and inflammatory conditions. Additionally, we explore the formation of tertiary lymphoid structures in chronic skin diseases as a window into studying B cell–ECM interactions. By integrating fundamental immunology with skin biology, we aim to identify key knowledge gaps and explore potential clinical implications of B cell–ECM interactions in dermatology and beyond.

## 1 Introduction

Resident B cells are yet an overlooked component of the skin immune system. The presence of B cells in healthy skin is a relatively new finding and is intriguing since the skin is, by definition, not a lymphoid organ where B cells typically reside [1]. They contribute to rapid pathogen recognition, antibody production and immune regulation in this peripheral tissue [2, 3, 4]. Remarkably, recent publications demonstrate that the skin, as an autonomous unit, can produce systemically protective IgG antibodies [5, 6]. These findings highlight the fundamental importance of B cells in skin and establish the skin as part of an autonomous adaptive immune organ. The extracellular matrix (ECM) in the skin, comprising both basement membranes and interstitial ECM, forms a complex microenvironment that significantly influences immune cell function [7, 8, 9]. The ECMs are not merely structural components but actively participate in shaping immune response [8]. Interstitial ECM and basement membranes occur within secondary lymphoid organs (SLOs), namely, lymph nodes and spleen [10, 11]. Recent studies indicate similarities in the proteome of the dermal and lymphoid ECM [12]. This could suggest close immune‐supportive functions of the dermal ECM.

The potential interaction of the ECM with B cells is—in part—maintained by integrins. Integrins are heterodimeric transmembrane glycoprotein cell‐surface receptors. They are the principal cell‐surface receptors that convey ECM instructions to intracellular cell fate responses, as evidenced by skin diseases caused by genetic ECM deficiency [13]. In terms of immune function, the integrin‐mediated signalling from the cell exterior to the cell interior appears to be crucial in shaping various aspects of B cell biology. This pathway significantly influences B cell destiny, maturation, longevity and overall functionality [14, 15, 16].

The interactions between B cells, integrins and the ECM in the skin context remain largely unexplored, presenting a frontier in immunological research. These interactions likely play pivotal roles in various physiological and pathological processes, including wound healing and inflammatory skin conditions such as hidradenitis suppurativa (HS) [1, 17]. Both wound healing and HS involve ECM turnover, which may significantly affect B cell function through altered integrin‐mediated signalling. Understanding the specific mechanisms of B cell–ECM interactions in the skin and the role of individual ECM proteins in modulating B cell behaviour could provide valuable insights into skin immunity. This knowledge may lead to novel therapeutic approaches for skin disorders and enhance our comprehension of the skin's immune system.

In this review, we synthesise current knowledge about the interactions between B cells, integrins and the ECM in the context of skin immunity. While recent studies have shed light on the presence and diversity of B cells in the skin, as well as the importance of ECM components and integrin signalling in immune function, there remains a significant gap in our understanding of how these elements interact specifically in peripheral organs like the skin. We aim to highlight critical areas where further research is urgently needed, including the role of individual ECM proteins in modulating B cell behaviour, the specific mechanisms by which skin‐resident B cells interact with the local ECM and the potential implications of these interactions for skin diseases.

## 2 Skin ECM Molecules: Direct Inducers of Immune Response, With Overlaps to SLO

The ECM is an organised network of structural and signalling proteins. It can be broadly categorised into two main components: the cell‐distal interstitial ECM and the cell‐adjacent basement membranes. The interstitial ECM is load bearing, occupying the spaces between cells, whereas basement membranes are specialised sheet‐like ECM structures that directly underlie epithelial and endothelial cell layers and surround muscle, fat and nerve tissues [7]. The ordered arrangement of both interstitial ECM and basement membranes, along with their structural and signalling protein components, is crucial for multicellular life [7]. The skin's architecture consists of three distinct layers: the epidermis sitting on a basement membrane, the dermis and subcutaneous fat tissue embedded in an interstitial ECM. A primary function of the skin is to serve as a defensive barrier against external threats. This protection is achieved through a combination of physical barriers, bioactive molecules and a complex network of resident immune and non‐immune cells, along with various ECM skin structures [18].

The skin's primary function is predominantly viewed as a barrier, with immune functions being secondary, while SLOs are specialised for immune cell interactions [18, 19, 20]. The ECM composition in SLO and the skin shares notable similarities. Both can form organised structures with separate B cell and T cell zones. In SLOs, these are inherent to their structure, while in the skin, they can form as tertiary lymphoid structures (TLS) during inflammation [21]. Interestingly, recent findings demonstrate that TLS can also assemble in healthy skin. They were identified adjacent to hair follicles, where they respond to the resident skin microbiome and generate systemic protective antibodies against these cutaneous commensals [5, 6]. The T cell zone conduits in SLO have been reported to contain the specific components laminin‐411, laminin‐511 and collagen VI [22, 23]. Conduits within SLO B cell zones, known as follicles, contain ECM components with relatively selective tissue distribution such as laminin‐332, collagen VII and cochlin [12, 24, 25]. The collectively aforementioned ECM molecules, laminin‐511, laminin‐322, collagen VI and collagen VII, which are found in conduits, are also present in the skin [26]. Nevertheless, little is currently known about their influences on adaptive immunity in the skin.

The ECM and the communication between different types of immune cells are crucial in controlling and modulating the immune responses [11, 12, 14, 24, 27, 28]. First, the lymphoid ECM provides a structural framework for immune cells to reside and interact in, while also serving as a reservoir for various signalling molecules that influence immune cell behaviour [11, 14, 29]. Importantly, however, the ECM itself is cell instructive and may influence immune cell fate and reactivity [30]. Abnormally expressed or deposited ECM molecules can affect the activation, differentiation and survival of immune cells [31]. Some of these molecules are specifically digested by enzymes, including matrix metalloproteinases (MMPs) [31]. This breakdown results in biologically active peptides that can either attract immune cells or modify their behaviour [8]. Research has shown that when collagen I is broken down by MMP8 and MMP9, it mimics the effects of the chemokine CXCL8 in attracting neutrophils to sites of lung inflammation [32]. These breakdown products can be detected in lung fluid samples from people with obstructive pulmonary diseases, suggesting they could potentially serve as a biomarker or therapeutic target for inflammatory diseases characterised by neutrophil involvement [33]. Additionally, products from the breakdown of elastin have been found to attract monocytes in chronically inflamed lungs [34, 35, 36]. While these processes have primarily been studied in lung inflammation models, they are likely relevant to other tissues where these molecules are upregulated during inflammation, such as the skin.

The way immune cell activation occurs through ECM molecules is still elusive. Some research indicates that Toll‐like receptors (TLRs) may play a key role as mediators [31]. TLRs identify specific molecular patterns linked to pathogens or tissue damage, including lipopolysaccharides (LPS) [37]. Findings suggest that either whole ECM molecules such as biglycan or certain elements or fragments of the ECM might also activate TLRs [38]. Typically, these fragments are generated by enzymes in inflamed tissues and are often found in the interstitial matrix [37]. Tenascin C, for example, binds to TLR4 on macrophages and synovial fibroblasts, leading to the production of pro‐inflammatory cytokines and resulting in inflamed synovium [39]. In mice lacking tenascin C, protection against this type of synovitis is observed [39]. Moreover, the spliced exon encoding type III of cellular fibronectin can activate TLR4 [40].

In summary, the ECM of SLO and the skin have some overlap. ECM molecules and ECM‐derived products can directly influence immune cells, via TLR engagement, possibly playing a key mediator role. However, the overall interactions of these components are not well understood, especially regarding potential interactions with B cells. Besides TLRs, integrins are also potential partners for such interactions [41]. The following section will explore current knowledge about integrins as cell surface molecules and their interactions with the ECM and B cells in more detail.

## 3 Integrins: Key Mediators to Orchestrate ECM B Cell Interaction

Integrins consist of an α and a β subunit. There are 18 α and 8 β subunits. In total, not all combinations exist, but rather 24 heterodimers are known [42]. They act as bidirectional communication channels, relaying signals from within the cell to the external environment (inside‐out signalling) and also transmitting cues from the ECM back into the cell (outside‐in signalling) [42]. A minimum of 12 integrin varieties are exhibited across diverse leucocyte and platelet cells (reviewed in [43]). In terms of immunity, the outside‐in signalling pathway mediated by integrins seems to play a particularly important role in determining the fate, development, survival and function of B cells (Table 1) [14, 15, 16].

**TABLE 1:** Summary of key integrins discussed in this review, their ECM interaction partners and their functions in B cells. Additionally, their known expression in skin and/or SLO is also provided.

Integrin	Ligand(s)	Function regarding B cells
α4β1	Fibronectin	Maintains MZ B cell population, essential for B cell compartmentalisation, critical for organisation within SLOs, mediates B_reg_ migration to inflamed skin, promotes B cell migration along with Il‐4 and facilitates lymphocyte motility
α5β1	Fibronectin	B cell migration
α6β1	Laminin‐511/521; Laminin‐311/322	Facilitates pro‐survival signalling in developing B cells, supports long‐term survival, promotes cell growth and survival in germinal center B cells, supports MZ B cell development from naïve B cells and enhances antibody response capabilities

### 3.1 Maintenance

**FIGURE 1:**
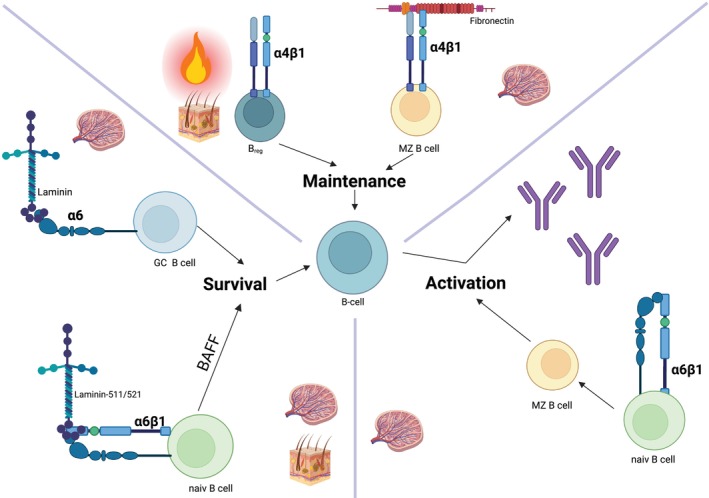
Integrin‐mediated B cell functions: (1) Maintenance in SLO: α4β1 interacts with the ECM, particularly fibronectin and maintains MZ B cell populations in the spleen, organising B cell compartmentalisation in SLO. B_regs_ migrate to inflamed skin via the integrin α4β1. (2) Survival: α6β1 integrin binds laminin‐511/521 in skin and SLOs. It facilitates pro‐survival signalling in developing B cells, partially mediated via BAFF in spleen and skin, supporting long‐term survival. GC B cells exhibit higher α6‐integrin expression, which lead to cell growth and survival through laminin binding. (3) Activation: α6β1 promotes their differentiation from naive B cells and enhances antibody response capabilities.

**FIGURE 2:**
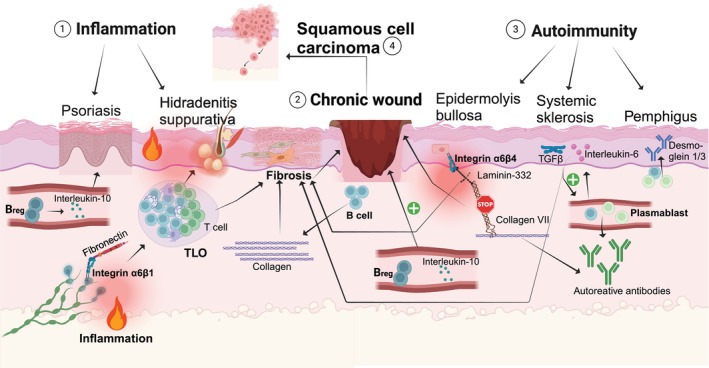
B cells in skin pathology: Interconnections between inflammation (1), chronic wounds (2) and autoimmunity (3), leading to squamous cell carcinoma (SCC) (4). Inflammation (1): B cells enter the skin through integrin α6β1‐mediated migration enhanced by fibronectin. They can take on different tasks: In psoriasis, B regulatory cells (B_regs_) produce anti‐inflammatory Interleukin 10. In contrast, in HS, TLS form in the dermis, containing B and T cells, which contribute to inflammation and fibrosis. This can lead to chronic wounds. Chronic wounds (2): B cells have a dual function in wound healing. They contribute to wound healing by promoting fibroblast synthesis of ECM molecules such as collagens and are key players in anti‐inflammatory responses via B_reg_‐produced interleukin 10. Autoimmunity (3): B cells contribute to pathology through both systemic and local mechanisms: In dystrophic epidermolysis bullosa (DEB), the absence of collagen VII disrupts interactions with laminin‐332 and integrin α6β4 potentially via altered growth factor bioavailability causing B cell dysregulation. DEB also results in chronic wounds (3). In systemic sclerosis, autoreactive antibodies are produced by B cells in the skin, where B cells mediate fibrosis through TGF‐β and interleukin 6. In pemphigus vulgaris, in addition to systemically acting B cells, resident skin B cells locally produce desmoglein 1/3 antibodies correlating with disease progression. All three conditions inflammation (1), chronic wounds (2) and autoimmunity (3) share common pathways to SCC development (4): altered ECM composition, immune system dysfunction and chronic inflammation.

Like in lymph nodes, the spleen exhibits a compartmentalised structure with distinct zones for T cells and B cells, fashioned through an intricate network of conduits. Its architecture can be broadly divided into two principal regions. Firstly, the white pulp, where T cells congregate within the lymphatic periarterial sheath, while B cells are mainly found in the lymphoid follicles. Secondly, the red pulp, which harbours a diverse array of resident and migratory cell populations [41, 42]. The marginal zone (MZ) encircles the T and B cell regions within the white pulp [44]. SLOs have well‐defined B cell subsets with known functions (e.g., MZ B cells, germinal center [GC] B cells). The α4β1 integrin, which can interact with the ECM among others via fibronectin, in particular, is essential for maintaining MZ B cell populations, as demonstrated by depletion studies in mice [15] (Figure 1, Table 1). This finding underscores the critical role played by the α4β1 integrin in regulating the compartmentalisation and organisation of B cells within the peripheral lymphoid organs [15]. The skin ECM likely plays a vital role in retaining B cells within the dermis. For example, interleukin‐(IL)10‐positive regulatory B cells (B_regs_), which help mitigate inflammation in the skin, have been shown to migrate via α4β1 integrin [45, 46] (Figures 1, 2). This suggests that integrin‐mediated interactions with the skin ECM may be essential for the localisation and function of such regulatory B cells. Recirculating B cells require specific signals to enter the tissues. Besides chemokines, integrins are crucial in this process. Indeed, IL‐4 and fibronectin have been shown to promote B cell migration (Figure 2). This could be inhibited by antibodies specific for α4β1 and α5β1 integrins, indicating a role for integrin‐ECM interactions in lymphocyte motility. The number of integrins on highly mobile B cells does not increase, but their avidity for ECM ligands is believed to be enhanced, facilitating easier movement [47].

### 3.2 Survival

The α6 integrin‐subunit is found in only two heterodimeric combinations: with β1 and β4 [48]. The α6β1 integrin on B cells binds to laminins and in skin predominantly to laminin‐511/‐521 and laminin‐311/332 [14, 49, 50]. This integrin facilitates pro‐survival signalling from laminin‐511/‐521 in developing B cells along with B cell activating factor (BAFF) (Figure 1, Table 1). GC B cells exhibit a six‐fold higher expression level of the α6‐integrin subunit compared to naive, non‐GC B cells within both Peyer's patches and the spleen [16]. The A20 B cell line, modelling GC B cells, highly expresses the integrin α6A isoform. The α6‐integrins on A20 and GC B cells form functional laminin‐binding receptors. Notably, anti‐α6 antibodies block A20 cell adhesion to laminin and increase the S‐phase population, implying α6‐integrin roles in cell growth and survival. Drawing insights from SLOs, integrin α6β1 on B cells binds various laminin isoforms, facilitating pro‐survival signalling and supporting the development and survival of specific B cell subsets [14, 49, 50] (Figure 1).

### 3.3 Activation

Integrin α6β1 is also expressed on MZ B cells, where it supports MZ B cell development from naive B cells in SLO, promoting their long‐term survival and antibody‐response capabilities [14] (Figure 1). In addition, laminin‐523 has been described to appear in the follicular border in murine lymph nodes [30]. At this position, it helps support B cell immune responses by guiding and regulating the activity of stromal cells, T‐follicular helper cells and dendritic cells [30]. The activities were described to be integrin α6β1‐dependent. This is likely a composite effect of interactions with other laminin isoforms, as laminin‐523 should only limitedly interact with integrins due to the absence of a glutamic acid in the laminin γ3 chain needed for integrin binding [51].

In summary, integrins like α4β1 and α6β1 mediate critical interactions with ECM proteins, regulating maintenance, survival and activation of distinct B cell subsets (Figure 1). However, their specific functions in skin B cell biology require further investigation. In the next section, we will explore the clinical significance of B cell integrin ECM interactions in dermatology.

## 4 Clinical Outlook in Dermatology

Null mutations for the ICAM receptor αL or β2 integrin subunits display a similar phenotype in mouse [52] and in humans. In humans, leukocyte adhesion deficiency is characterised by recurrent infections, impaired leukocyte adhesion and compromised migration to sites of inflammation and infection within the skin [53]. Blocking antibodies targeting integrin α4β1, such as natalizumab, and integrin αLβ2, such as efalizumab, have been employed in the treatment of multiple sclerosis and psoriasis, primarily affecting T cell immune response [54, 55]. These therapeutic approaches demonstrate the importance of integrin‐mediated interactions in T cell‐driven immune response. While integrin ECM interactions are well‐characterised, focusing on T cells in skin cancer, autoimmune disorders, and inflammatory conditions, the specific role of B cells within these interactions remains under investigated. Traditionally, the skin is not considered a prime site for B cell differentiation and antibody secretion. In this perspective, pathogenic autoantibodies generated in lymphoid tissues would enter the bloodstream and subsequently diffuse into the skin, where they would contribute to the development of skin diseases [1]. The niche of resident B cells is currently underrepresented in dermatology, as they are not considered major players in many diseases. However, emerging evidence suggests that B cell integrin ECM interactions could play a vital role in cutaneous immune response and homeostasis (Figure 2). B cells in the skin play important roles in immune defence. They can identify common features of pathogens, like pathogen‐associated molecular patterns (PAMPs) [2]. Their presence in the skin serves as a rapid defence mechanism against pathogens, particularly after barrier breaches [1]. Plasma cells generate protective IgM and IgA antibodies. These antibodies are transported across epithelial barriers and coat mucosal surfaces, helping to prevent invasion and maintain immune protection [3, 4]. Emerging research demonstrates the skin's unexpected ability to independently generate systemic protective IgG antibodies [5, 6].

### 4.1 Autoimmunity

The roles of ECM and B cells have been reviewed separately in the context of autoimmune skin diseases, but there is a lack of approaches that integrate and combine the knowledge from these two areas [56, 57]. While systemic autoantibodies play a crucial role in various skin diseases, recent evidence suggests that resident B cells in the skin may contribute significantly to local pathology. In a lupus mouse model with B cells lacking the capacity to produce antibodies, a lupus‐like disease still developed. In contrast, mice completely depleted of B cells did not develop the disease [58]. In pemphigus, the presence of circulating anti‐Desmoglein‐1/3 (Dsg1 and Dsg3) autoantibodies in individuals without skin lesions indicates that additional factors, potentially including local immune responses, influence disease manifestation [59]. Yuan and colleagues recently revealed that autoreactive Dsg1 and Dsg3 specific antibody‐secreting CD19+ B cells and CD138+ plasmablasts are more prevalent in lesional skin compared to healthy skin in pemphigus patients [60]. This finding suggests that autoreactive cells may play a direct pathogenic role at the site of inflammation.

In systemic sclerosis, elevated levels of pathogenic B cells are found in the blood. These B cells contribute to fibrosis through several mechanisms, including direct cell‐to‐cell contact, secretion of IL‐6 and secretion of TGF‐β. The secretion of TGF‐β leads to increased ECM synthesis, including collagens. IL‐6 secretion, in turn, stimulates B cell proliferation and autoantibody production, creating a feedback loop that further promotes the disease process [61]. B cells are also found in the skin of systemic sclerosis patients. Studies have shown that the degree of CD20+ B cell and CD138+ plasmablast infiltration in the skin correlates with worsening skin scores [62]. However, there is limited information about the specific function of these B cells in the skin. Additionally, we lack a clear understanding of which ECM proteins help these B cells remain in the skin tissue.

DEB is an inherited disorder characterised by fragile skin. Pathogenic *COL7A1* gene variants cause DEB. *COL7A1* encodes collagen VII, which forms anchoring fibrils that promote firm attachment of the epidermal basement membrane to the interstitial dermal papillary ECM [63]. Loss of functional collagen VII in the dermal ECM leads to blister formation upon minimal mechanical stress [64]. The tissue injury and inflammation associated with DEB exacerbate the progressive nature of the condition [65]. Importantly, collagen VII is not only present in the skin but is also found in the ECM of B cell follicles in SLO. Collagen VII, as in skin, in the spleen forms a hypothetical interaction axis with laminin‐332–integrin α6β4 [12, 66]. Recent studies have suggested that some degree of innate immune dysfunction is associated with DEB [12]. On the one hand, DEB appears to be linked to B cell autoimmunity [67, 68, 69, 70, 71, 72]. Conversely, it can also result in under‐reactive immunity in regard to impaired immune responses against bacterial infections [12]. These findings suggest that the absence of collagen VII in SLO disrupts immune homeostasis, leading to both autoimmune manifestations and compromised immune defences. Since the major interaction partners of collagen VII, collagen IV and laminin‐332, are present in both SLOs and skin, it could imply B cell regulatory functions of collagen VII also in skin.

### 4.2 Wound Healing

Skin wounds are categorised as acute or chronic based on their development and outcomes [73]. Acute wounds follow a sequence of molecular processes that lead to structural recovery. In contrast, chronic wounds remain unresolved, marked by ongoing issues like persistent inflammation, recurring infections and tissue death [74]. The healing process of acute wounds is a dynamic, well‐coordinated process that unfolds in four distinct phases: haemostasis, inflammation, proliferation and remodelling [75]. The inflammatory phase provides valuable insights into how ECM interactions with immune cells function in a regulatory manner [75]. However, B cell ECM interactions during wound healing remain understudied, leaving a gap in our understanding of their potential interplay in this process [76].

Specific ECM components play crucial roles in guiding the immune response. Collagen III‐rich collagen fibres and fibronectin in particular serve as scaffolds that direct inflammatory cells to the wound site. This targeted migration is essential for initiating and modulating the healing process [75, 77]. During wound healing, keratinocytes interact with the ECM through various integrins to facilitate migration and re‐epithelisation. Initially, β1 integrins interact with fibrinogen, fibronectin and collagens in the provisional ECM. As healing progresses, keratinocytes deposit laminin‐332 and switch to expressing integrin α6β4 [66]. The laminin‐332/integrin α6β4 interaction is crucial for directed migration of keratinocytes, reassembly of the dermal–epidermal junction zone and establishment of stable epidermis [78, 79]. Another key ECM component of wound healing is collagen VII, which in addition to securing the mature epidermal basement membrane to the papillary ECM through forming anchoring fibrils, also actively instructs re‐epithelialisation and dermal healing [66].

B cells significantly influence normal wound healing [1]. They reduce the expression of inflammatory molecules and increase proteins associated with proliferation, tissue remodelling and protection from oxidative stress [80]. They localise to wounds and influence the healing process through the production of cytokines such as IL‐6, IL‐10 and TGF‐β [81]. Mice with overexpressed CD19, a B cell receptor co‐receptor, exhibit accelerated wound healing, while CD19‐deficient mice show delayed healing [81]. The application of splenic B cells to wounds has been shown to accelerate healing in both wild‐type and diabetic mice, further supporting the role of B cells in acute and chronic wound repair [82]. Additionally, B_regs_ secreting IL10 have been found to play a significant role in wound healing [83]. B_regs_ suppress immune responses and promote tissue repair by producing anti‐inflammatory cytokines and interacting with other immune cells [84].

Conversely, B cells have also been found to exhibit pro‐fibrotic and pro‐inflammatory properties in wound environments. In vivo experiments on mice revealed that up to 30% of B cells applied to skin wounds acquired an immunomodulatory phenotype after adoptive cell transfer [80]. Moreover, researchers found that B cells, particularly plasmablasts, from IgG4‐RD patients can directly stimulate fibroblasts to increase collagen production [85]. Surprisingly, these B cells can also produce collagen themselves and participate in organising the extracellular matrix [85, p. 4]. This suggests that B cells have a more direct role in ECM modulation than previously thought.

### 4.3 Inflammatory Skin Diseases

B cells in inflammatory skin diseases have been extensively reviewed by Debes et al. [1] The quantity of B cells is increased in affected skin lesions compared to healthy skin samples across various inflammatory conditions, such as psoriasis [86], atopic dermatitis (AD) [87] and HS [17, 88]. They can cluster in inflamed skin in TLS which, similar to SLO, contain separate B cell and T cell zones in combination with follicular dendritic cells [21, 56] (Figure 2). Their function appears to be variable. On the one hand, they can act in an anti‐inflammatory manner, which manifests clinically in the aggravation of psoriasis under rituximab treatment [89, 90]. This immunosuppressive function is primarily mediated by B_regs_ through their secretion of IL‐10 [91, 92]. B_regs_ represent the principal B cell population in lesional psoriatic skin [91, 92]. On the other hand, they can also act to contribute to inflammation as suggested by the successful use of B cell depletion with rituximab in HS and AD cases [93, 94]^(p20)^.

A study investigated the impact of BAFF on B/plasma cells in HS lesions. They saw that B cells in HS lesions cluster in TLS‐like structures, maintained by 67 potential molecules associated with the BAFF pathway, including several integrin‐related molecules like CD37 and CD53 [88]. These proteins play crucial roles in B cell and plasma cell function, particularly in their interaction with ECM. CD37 organises and stabilises integrin α4β1at the cell membrane, promoting cell spreading, migration and binding to ligands like VCAM‐1 and fibronectin in the ECM [95, 96]. In addition to supporting integrin α4β1 in binding to VCAM‐1, the CD37 molecule is also essential for sustaining the viability and survival of plasma cells [97]. CD53 complexes with integrins and stabilises L‐selectin surface expression, which may facilitate B cell trafficking into HS lesions [98, 99]. Yu et al. demonstrated that TLS formation in HS is spatially localised near fistular tracts, correlating with increased B cell infiltrate, predominantly plasma cells (CD38+), which produce antibodies against keratinocytic proteins [21]. Additionally, increased numbers of CD19+ B cells can be detected, while memory B cells, naive B cells and GC B cells are less frequent, along with a small population of regulatory B cells [21]. The B cell subpopulation profile observed in HS mirrors that seen in autoimmune skin diseases (such as pemphigus and systemic sclerosis as shown above), challenging the ongoing debate about whether HS should be classified as an autoinflammatory or autoimmune disease. The TLS formation and antibody production in HS is promoted by CXCL13‐expressing fibroblasts of likely reticular origin, which makes the TLS microenvironment similar to SLOs [21]. A significant aspect of HS pathology is the alteration of the ECM, characterised by fibrosis and tissue remodelling [100, 101, 102]. These changes contribute to the disease's chronic and progressive nature [100, 101, 102]. However, the specific impact of these ECM modifications on B cell infiltration and the development of TLS in HS remains unexplored, presenting an important area for future investigation.

In summary, several BAFF pathway elements identified seem to modulate B/plasma cell functions like migration, adhesion and trafficking through interactions with integrins and their ligands in the ECM, potentially contributing to their recruitment into HS skin lesions. Therefore, we believe that HS can serve as an excellent model to study B cell integrin ECM interactions, which could provide further insights into the functions of B cells in the skin. Potentially, these findings could be translated to the function of ECM in SLO.

### 4.4 Squamous Cell Carcinoma

All three conditions discussed above (autoimmunity, wound healing and inflammation) share a common risk: in chronic wounds, such as DEB wounds, leg ulcers and chronic HS lesions, the risk for development of aggressive SCC is significantly elevated [85, 86, 87, 103, 104, 105, 106]. This phenomenon can be largely attributed to the altered composition and function of the ECM in these chronically damaged areas. This phenomenon could potentially be explained by the concept of a localised immune dysfunction in areas of chronically damaged skin because of altered ECM composition. In regions of chronic damage, the ECM undergoes significant changes, leading to a localised immune dysfunction. These alterations in the ECM composition compromise normal immune surveillance mechanisms, creating an environment that is both less restrictive to tumour formation and less supportive of proper immune cell function [106]. The modified ECM affects various aspects of the local tissue environment:

1. Immune cell infiltration and function: The altered ECM can impede the normal movement and activation of immune cells, weakening the local immune response [107].
2. Lymphatic drainage: A key factor contributing to this increased risk is the stasis of lymphatic drainage, which is a known complication in HS and chronic ulcers. This impaired lymphatic flow, partly due to ECM changes, hinders the normal movement of immune cells into and out of the affected area [105, 108].
3. Tumour cell behaviour: Changes in the ECM can provide a more permissive environment for tumour cell proliferation, invasion and eventual metastasis [106, 109].

It is important to note that SCCs arising in chronic wound environments are distinctly different from those developing in normal skin. Wound‐associated SCCs tend to be more aggressive and have a higher likelihood of metastasising. The ECM changes observed throughout the body in the aforementioned conditions not only affect the local wound environment but also tend to promote a state of chronic systemic inflammation [85, 86, 87]. A systemic inflammatory state, coupled with impaired immune system function (both influenced by ECM alterations), may create a more permissive environment for tumour metastasis. The compromised immune surveillance, both locally and systemically, appears to facilitate the spread and establishment of tumour cells in distant sites, contributing to the increased metastatic potential of these wound‐associated SCCs.

### 4.5 Major Open Questions

There are three major questions we want to highlight.

#### 4.5.1 How Do B Cell ECM Interactions in the Skin Compare to Those in SLOs?

Both skin and SLO contain similar ECM components, including laminin‐511, laminin‐332, collagen VII and fibronectin. In SLOs, these ECM components form specialised structures like conduits that support B cell localisation and function. Skin B cells interact with the ECM through integrins similar to SLO B cells, but their specific roles in the skin are less well‐characterised. The skin ECM likely plays a crucial role in retaining B cells within the dermis, but the exact mechanism and functional implications require further investigation.

#### 4.5.2 What Role Do B Cells Play in Wound Healing and How Might This Be Influenced by ECM Interactions?

B cells significantly influence wound healing by reducing inflammatory molecules and increasing proteins associated with proliferation and tissue remodelling. They produce cytokines such as IL‐6, IL‐10 and TGF‐β that contribute to the healing process. B cells interact with the ECM through integrins, which may guide their localisation and function in wounds. However, B cells can also exhibit pro‐fibrotic properties, directly stimulating fibroblasts to increase collagen production and even potentially producing or modulating collagens themselves. Thus, more studies are needed to address the contextuality of events.

#### 4.5.3 How Do ECM Alterations in Chronic Skin Conditions Contribute to Increased Cancer Risk?

In chronic skin conditions, ECM alterations lead to localised immune dysfunction, creating an environment more permissive to tumour formation. These changes impede normal immune cell infiltration and function, disrupt lymphatic drainage and provide a more favourable environment for tumour cell proliferation and invasion. The altered ECM also contributes to a state of chronic systemic inflammation, which, coupled with impaired immune function, may facilitate tumour metastasis. This may explain why SCCs arising in chronic wound environments tend to be more aggressive and have a higher likelihood of metastasising. However, this notion needs further experimental support.

## 5 Conclusion and Perspectives

The ECM plays a crucial role in both SLO and the skin, providing structural support and facilitating immune cell interactions. B cells, while fundamental for systemic immune homeostasis, are less commonly considered but are increasingly recognised as important in healthy skin. Integrin‐mediated interactions between B cells and the ECM, particularly through integrins α4β1 and α6β1, are critical for B cell localisation, development and function in both environments (skin and SLO). These interactions influence various aspects of skin health, including wound healing, autoimmune responses and inflammatory conditions. In diseases like HS, B cells form TLS, highlighting their potential role in chronic inflammation. ECM influence on B cell behaviour in the skin remains an underexplored area, with potential implications for understanding and treating skin disorders. Chronic inflammation and ECM alterations in conditions like epidermolysis bullosa can lead to an increased risk of aggressive SCC, emphasising the importance of maintaining ECM homeostasis. Future research should focus on elucidating the specific roles of B cell ECM interactions in skin health and disease, potentially leading to novel therapeutic approaches in dermatology.

## Author Contributions

R.D., S.H., S.L., M.R., K.E. and A.N. wrote the paper. R.D. made the figures. R.D. and A.N. conceptualised the paper.

## Conflicts of Interest

The authors declare no conflicts of interest.

## Data Availability

The data that support the findings of this study are available from the corresponding author upon reasonable request.
